# Deglutition dynamics of patients with obstructive sleep apnea

**DOI:** 10.20407/fmj.2023-010

**Published:** 2024-02-15

**Authors:** Satoshi Ito, Yoichiro Aoyagi, Masatoshi Hirata, Miho Ohashi, Hitoshi Kagaya, Hiroya Inada, Ayami Kimura, Kazuki Shikano, Masamichi Kaneko, Takayuki Okano, Seiichi Nakata

**Affiliations:** 1 Department of Otolaryngology and Sleep Medicine, Fujita Health University, School of Medicine, Nagoya, Aichi, Japan; 2 Department of Rehabilitation Medicine, Nippon Medical School, Bunkyo-ku, Tokyo, Japan; 3 Department of Clinical Laboratory, Fujita Health University Bantane Hospital, Nagoya, Aichi, Japan; 4 Department of Rehabilitation Medicine I, Fujita Health University, School of Medicine, Toyoake, Aichi, Japan

**Keywords:** Deglutition, OSA, EMG, Sleep

## Abstract

**Objectives:**

In the present study, we performed a detailed analysis of deglutitive dynamics during sleep in patients with obstructive sleep apnea (OSA) using a methodology developed by Sato et al. We hypothesized that the frequency of deglutition would decrease with increasing severity of OSA. The aim of this study is to clarify the involvement of deglutitive dynamics during sleep in OSA by investigating the correlations between deglutition and sleep parameters.

**Methods:**

This study included 30 adult patients with OSA. To analyze deglutition dynamics during sleep, surface electromyography recordings of the suprahyoid and thyrohyoid neck muscles, which are involved in deglutition, were performed simultaneous with conventional polysomnography. The “index of deglutition” was defined as the frequency of deglutition per hour of sleep. We examined correlations between this index and sleep parameters (apnea-hypopnea index [AHI], apnea index, hypopnea index, and lowest blood oxygen saturation).

**Results:**

By analyzing the obtained polysomnography and electromyography waveforms, we identified two deglutition patterns with and without respiratory arousal during sleep. We found a significant negative correlation between the index of deglutition in sleep stage 1 and the AHI, with a correlation coefficient of –0.48. (p=0.02)

**Conclusions:**

In the current study, we distinguished deglutition during sleep with and without arousal. In addition we discovered a significant negative correlation between the index of deglutition in sleep stage 1 and the AHI. This new finding will provide a platform for future research on OSA in aspiration pneumonia.

## Introduction

Deglutition, the process of transporting food masses into the gastrointestinal tract, involves complex movements. Maintenance of pharyngeal clearance through deglutition also plays an important role in airway defense.

Sleep is a physiological state in which consciousness is periodically lost and various vital activities, including physical activity, are diminished. However, external stimuli can easily lead to awakening. Little is known about deglutition during sleep.

In 1965, Lear et al. used pneumatic and sonic methods to measure the frequency of deglutition over a 24-hour period. They reported that healthy adults swallowed an average of 585 times per day, 180±55 times per hour during meals, 23.5±11.4 times per hour when not eating, and 5.3±1.7 times per hour during sleep. Moreover, they reported increased deglutition frequency on sleep onset and awakening, with a 20-minute period free from deglutition.^1^ Another study using the ultrasonic pulse Doppler method to examine deglutition dynamics during sleep was the first to report deglutition patterns in association with sleep stages. In that study, Lichter et al. reported an average of 5.8 deglutition per hour of sleep. They also reported that, on 33.3% of occasions, deglutition occurred during rapid eye movement (REM) sleep, while on 20.2% of occasions it occurred non-REM stage 1 and on 31.7% during non-REM stage 2. The researchers found that deglutition occurred almost exclusively in association with motor arousal and was most frequent during non-REM sleep stages 1 and 2 in healthy adults. However, many unresolved issues remain regarding deglutition during nocturnal sleep and the respiratory dynamics associated with deglutition.^2^

Furthermore, there have been few reports on the relationship between deglutition and obstructive sleep apnea (OSA), a major sleep disorder. OSA is characterized by sleep-disordered breathing accompanied by narrowing of one or more areas along the upper airway during sleep. Apnea is defined as cessation of breathing for at least 10 seconds, and is usually accompanied by sleep fragmentation (electroencephalographic arousals) and decreased oxygen saturation. Epidemiological studies estimate its incidence in the general population to be 2%–5%.^3^

In recent years, polysomnography (PSG) has been digitized, allowing more detailed examination of physiological functions during sleep. Taking advantage of this, Sato et al. investigated the dynamics of deglutition during sleep in greater detail by simultaneously recording surface electromyography (EMG) of the suprahyoid and thyrohyoid muscles on PSG. The average frequency of deglutition during sleep was low, at 2.4±1.0 per hour of actual sleep time, and the frequency of deglutition was associated with the sleep stage and decreased as sleep deepened.^4–6^

Conventional video endoscopy and video fluoroscopy are invasive and very difficult to perform during sleep. In addition, video endoscopy and video fluoroscopy are mainly used to assess the quality of deglutitive function and check for dysphagia; they are not suitable for obtaining quantitative data, such as the rate of deglutition per hour. In recent years, esophageal manometry, pharyngeal impedance, and EMG have been attracting attention as alternative testing tools. Ohashi et al. compared these tools and determined whether they can detect deglutition events. They reported that esophageal manometry and pharyngeal impedance were somewhat superior to the above-described tools.^7^ However, it has been reported that EMG can also adequately detect deglutition. Esophageal manometry requires the insertion of a manometer into the esophagus through the nasal cavity, which makes measurement during sleep difficult. Additionally, there is no machine that simultaneously records pharyngeal impedance and PSG data. Therefore, we decided to use EMG in this study.

In daily clinical practice, we encounter patients who develop aspiration pneumonia even though aspiration is not obvious in tests of deglutitive function. It is believed that such patients may develop aspiration pneumonia because of impaired deglutitive function during nocturnal sleep. Furthermore, it has been suggested that aspiration pneumonia may be associated with nocturnal deglutition and respiratory phase patterns.^3^ The purpose of this study is to further clarify the relationship between the severity of OSA and the frequency of deglutition using similar methods to those of Sato et al.

## Methods

### Participants

The participants were patients who visited the Department of Otolaryngology, Bantane Hospital, Fujita Health University from January 2022 to January 2023 and underwent PSG to diagnose sleep disorders. We explained the purpose of the study to all patients. All participants had a sufficient understanding of the research and provided written consent. Of the patients who met the study criteria, 30 for whom PSG and EMG data were available were enrolled. OSA was defined as an apnea-hypopnea index (AHI) ≥ 5.0. This study was approved by the Ethics Review Committee of Fujita Health University (approval number: HM20-088).

### Polysomnography and definition of sleep apnea

PSG was performed using the Embla N7000 system (Natus, Middleton, WI, USA). We recorded electroencephalography (EEG), eye movement, miter muscle EMG, mouth and nose temperature, nasal pressure, snoring sounds, electrocardiography, tibialis anterior EMG, arterial blood oxygen saturation (SpO_2_) chest and abdominal respiratory motion, and body position data.

Apnea and hypopnea events were determined on the basis of the international standards of the American Academy of Sleep Medicine (version 2.5),^8^ and the sleep stages of children and adults were determined by a clinical technologist who analyzed EEG arousal responses and respiratory events. The respiratory events were visually confirmed by an expert clinical laboratory technician according to predefined criteria.

We conducted standard PSG with various physiological electroencephalogram parameters (C4-A1, F4-A1, and O2-A1) of the reference electrode derivation principle, as well as electrooculography and chin/anterior tibial electromyography. Nasal airflow/mouth-breathing analysis was performed using a thermistor and pressure sensors, chest wall/abdominal motion was analyzed using a strain gauge, snoring sounds were analyzed using a microphone, oxygen saturation was analyzed by pulse oximetry, and electrocardiography was also performed in an electrically shielded room designed for sleep studies. We calculated the AHI using the “alternative substitution rule,” as well as the 3% oxygen desaturation index, arousal index, and number of periodic limb movements per hour according to the American Academy of Sleep Medicine manual for scoring sleep and associated events.^9^

The criteria for classifying respiratory events in adults are as follows: for apnea, the respiratory amplitude of the mouth/nose temperature sensor must decrease by >90% from the pre-event baseline and the duration must be >10 seconds; and for hypopnea, the respiratory amplitude of the nasal pressure must decrease by >30% from the pre-event baseline and the duration must be >10 seconds. Hypopneas were analyzed visually if the respiratory amplitude of the nasal pressure decreased by >30% from the pre-event baseline, the duration was >10 seconds, and the oxygen saturation decreased by >3% from the pre-event baseline or there was an arousal response.

### Surface electromyography

**Figure 1:**
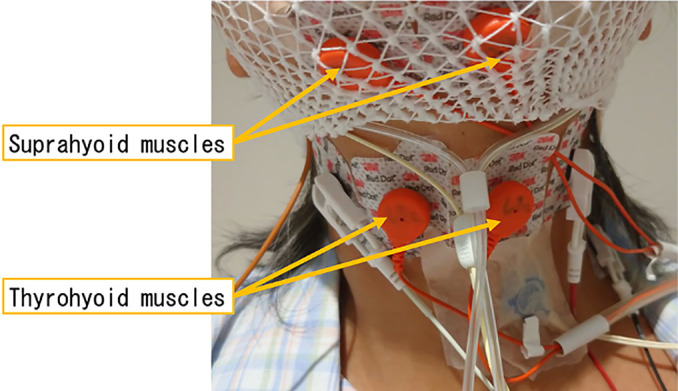
The surface electromyogram to the suprahyoid and thyrohyoid muscles

**Figure 2:**
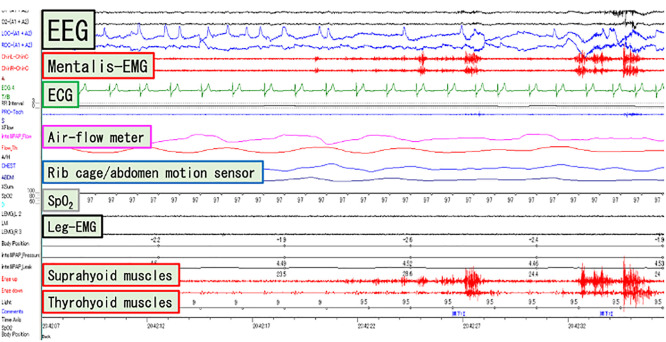
PSG EEG: Electroencephalogram, EMG: Electromyography, ECG: Electrocardiogram

Measurements were made by attaching EMG electrodes to the suprahyoid and thyrohyoid muscles (Figure 1) and simultaneously recording conventional PSG (Figure 2). In daily clinical practice, PSG typically does not measure neck EMG. However, in this study, we decided to record deglutition activity during sleep by adding neck EMG. Deglutition is a movement that propagates continuously from the pharynx to the esophagus. Therefore, a continuous waveform of the suprahyoid and thyrohyoid muscles indicates continuous propagation, i.e., deglutitive movement. An increase in the EMG potential of the suprahyoid muscles followed by an increase in the EMG potential of the thyrohyoid muscle was taken to indicate deglutition. Similar waveforms have been considered to reflect deglutition in previous reports.^3,5,6^

The frequency of deglutition during sleep time is determined on the basis of the surface EMG waveform. The “index of deglutition” is calculated by dividing the total frequency of deglutition by the total sleep hours. The purpose of this study was to examine the relationship between the severity of OSA and deglutition. Therefore, we examined the correlations between the index of deglutition and various sleep parameters (the AHI, apnea index [AI], hypopnea index [HI], and lowest SpO_2_).

### Statical analysis

Continuous data are expressed as mean±standard deviation, and categorical data as numbers. All statistical analyses were conducted using SPSS for Windows software (version 22.0; SPSS Inc., Chicago, IL, USA). Pearson correlation analysis and simple regression were used to assess the relations between the frequency of deglutition and sleep parameters. The statistical significance of correlation coefficients and regression lines obtained from the analysis of deglutition frequency and sleep parameters was determined by the t-values. A p-value <0.05 was considered statistically significant.

## Results

The participants were 30 adult patients with OSA. The male to female ratio was 23:7, and the mean age was 51.2±16.3 years. The mean body mass index was 28.3±5.46, the mean AHI was 44.6±23.0, and the lowest SpO_2_ was 79.5%±5%.

**Figure 3:**
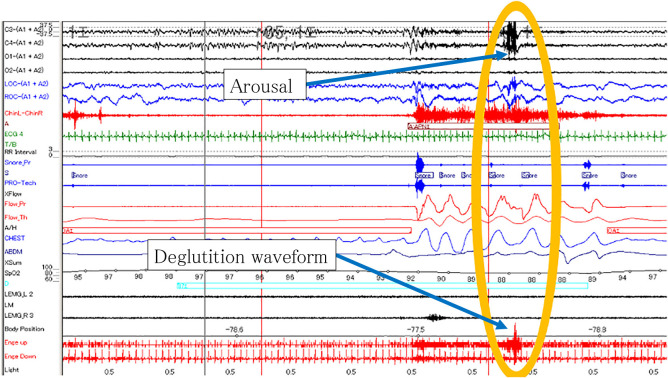
Deglutition with respiratory arousal

**Figure 4:**
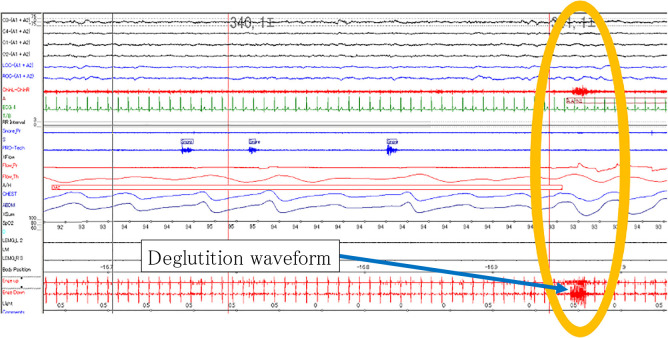
Deglutition without respiratory arousal

By analyzing the PSG and EMG waveforms, we discovered two novel deglutition patterns during sleep: deglutition followed by respiratory arousal (Figure 3), and deglutition unrelated to respiratory arousal (Figure 4). These two patterns of deglutition were analyzed separately.

For all sleep stages, the mean index of deglutition was 3.4±2.5 with arousal, 2.3±1.5 without arousal, and 5.7±3.1 for both deglutition patterns combined. The mean index of deglutition for sleep stage 1 was 5.4±3.7 with arousal, 4.3±3.5 without arousal, and 9.7±6.1 for both deglutition patterns combined, while for sleep stage 2 it was 1.2±3.1 with arousal, 0.7±1.4 without arousal, and 1.9±3.7 for both deglutition patterns combined. Finally, the mean index of deglutition for REM sleep was 4.7±6.5 with arousal, 1.6±2.7 without arousal, and 6.3±6.7 for both deglutition patterns combined (Table 1).

**Figure 5:**
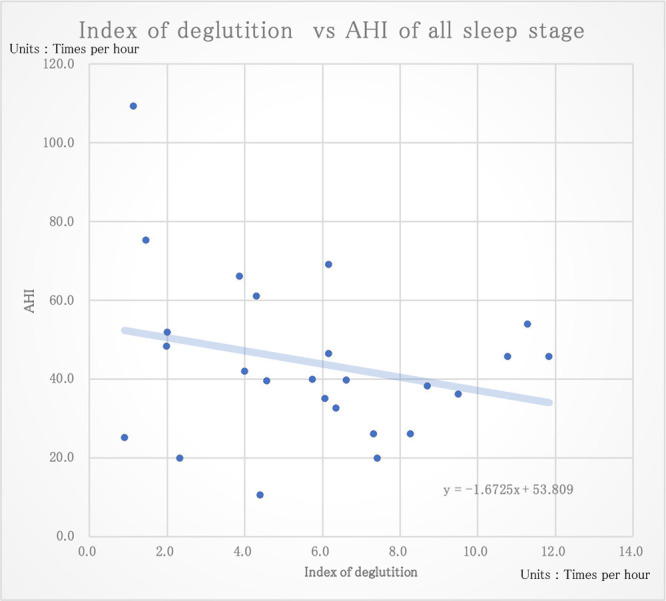
The correlation coefficient between the index of deglutition and AHI of all sleep hours

**Figure 6:**
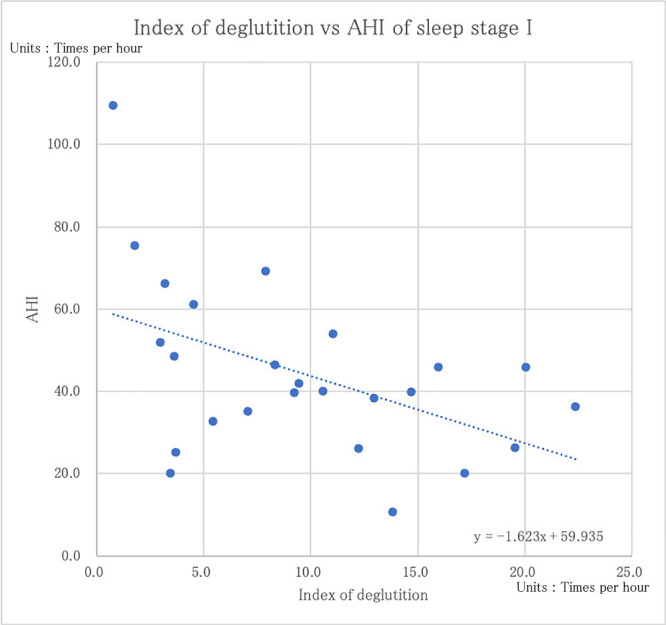
The correlation coefficient between the index of deglutition and AHI of sleep stage I

The correlation coefficients between the index of deglutition for all sleep stages and the various sleep parameters are shown in Table 2. For all sleep stages, there was a negative correlation between the AHI and the index of deglutition with arousal, without arousal, and for both deglutition patterns combined (–0.25, –0.17, and –0.24, respectively; Figure 5) However, the correlations were not statistically significant (p=0.22, 0.41, and 0.24, respectively). The correlations between the AHI and index of deglutition in each sleep stage were also examined. In Stage 1, there was a weak but statistically significant negative correlation of –0.48 (p=0.02; Figure 6), but there were no significant correlations for any other sleep stages or sleep parameters (Table 3).

## Discussion

Sato et al. reported that deglutition during sleep in healthy adults was associated with spontaneous EEG arousals. The normal deglutition-breathing pattern was found to involve exhalation, followed by deglutition and then by inhalation. In contrast, patients with OSA exhibited inspiration, followed by deglutition and by then exhalation.^10^ These findings suggest an antiphase relationship between the normal deglutition-breathing pattern observed in healthy adults and the deglutition-breathing pattern of OSA patients during sleep. This is supported by reports that the deglutition-breathing pattern of OSA patients treated with continuous positive airway pressure is similar to that of healthy adults.^6,11^

In this study, we focused on respiratory arousal and reported deglutition patterns rather than the deglutition-breathing pattern reported previously. We distinguished two different patterns of deglutition during sleep in OSA patients: deglutition associated with respiratory arousal and deglutition unrelated to respiratory arousal. Furthermore, we examined the correlations between the two deglutition patterns and the severity of OSA. The index of deglutition was defined as the rate of deglutition per hour of sleep. The index of deglutition for all sleep stages was 5.7±3.1 in our OSA patients, which was shorter than that of healthy adults. This result is largely consistent with a previous study.^3^ Moreover, the index of deglutition decreased as the sleep depth increased: in sleep stage 1, it was 9.7±6.1, slightly higher than in the previous study, while in stage 2 it was 1.9±3.7, slightly lower than in the previous study. Finally, for REM stage it was 6.3±6.7, slightly higher than in the previous study. These results indicate that the index of deglutition is related to sleep depth and may decrease as sleep deepens. This is presumed to be related to the cessation of various physiological actions as sleep depth increases in non-REM sleep. In REM sleep, the muscles are typically relaxed, so we assumed that deglutition would be less likely to occur compared with non-REM sleep; however, the index of deglutition was higher than expected. We speculate that the reason for this result is that the deglutition mechanism differs between REM sleep and non-REM sleep; that is, deglutition without respiratory arousal is more common in non-REM sleep than in REM sleep. The mechanisms are unknown at this stage, and we believe that this issue warrants future study.

We then examined the correlation between OSA severity and the index of deglutition, and we were able to demonstrate a statistically significant negative correlation between the index of deglutition of sleep stage 1 and the AHI. In addition, although not statistically significant, there was a weak negative correlation between the index of deglutition and the AHI for all sleep stages. However, there was no clear correlation between the AHI and any individual sleep stage. This suggests that the index of deglutition may decrease with increasing severity of OSA. Moreover, the index of deglutition may decrease more significantly when sleep is relatively shallow. This suggests that the index of deglutition during sleep is correlated with the severity of OSA when sleep is shallow, but the correlation disappears when sleep is deep. Thus, when comparing healthy adults and OSA patients, sleep depth differences may have an effect. Conversely, there is no difference between healthy adults and OSA patients in deep sleep, and we speculate that OSA patients may have deglutition and breathing abnormalities during shallow sleep.

Deglutition does not occur in sleep stage 3 and may occur only during relatively shallow sleep. However, in the present study, the frequency of deglutition in sleep stage 2 was very low, and it is possible that a correlation was not found because of the relative lack of samples. Furthermore, as already mentioned, REM sleep is different from non-REM sleep, and we believe that the relationship between breathing and deglutition may also differ depending on the sleep stage. Against this background, it is possible that the effect of deglutition in sleep stage 1 is stronger than that in other sleep stages; indeed, there were only weak negative correlations in the other stages.

In daily clinical practice, we encounter patients who develop aspiration pneumonia even without obvious aspiration in tests of deglutitive function. Studies have also reported that OSA patients have a 1.87-fold higher risk of being hospitalized for pneumonia compared with healthy adults.^12^ In addition, there have been reports of improvements in recurrent aspiration pneumonia in patients with OSA who had multiple episodes of aspiration pneumonia and received appropriate continuous positive airway pressure therapy.^11^ It is believed that such patients may develop aspiration pneumonia because of decreased deglutitive function during nocturnal sleep. In fact, deglutition during sleep is significantly reduced even in healthy adults, suggesting decreased pharyngeal-esophageal clearance.^4,13^ Sato et al. noted that deglutition during sleep is even less frequent in OSA patients. Respiratory effort tends to decrease intrathoracic pressure during episodes of upper airway obstruction in patients with OSA; as a result, Sato et al. speculated that a decrease in respiratory effort and intrathoracic pressure, along with disruption of the deglutition-breathing pattern, may decrease pharyngo-esophageal clearance and adversely affect aspiration pneumonia.^3^ In addition, the present study revealed a negative correlation between the severity of OSA and the index of deglutition. This suggests that, as OSA becomes more severe, the frequency of deglutition during sleep may decrease, leading to decreased pharyngoesophageal clearance. It also appears that this tendency may be more pronounced in situations where sleep is relatively shallow. Therefore, OSA may adversely affect pharyngoesophageal clearance and, depending on its severity, could also increase the risk of developing aspiration pneumonia.

## Limitations

This study had some limitations. First, the number of samples was too small to clarify the correlation between AHI and deglutition. Second, there was a sex bias in the overall sample. In the future, it will be necessary to include more female cases and eliminate sex bias. Third, EMG evaluates deglutition indirectly. For a more accurate evaluation, tools that directly evaluate deglutition should be used. Regardless, the tools used to assess deglutition are highly invasive and alternative options should be examined in the future.

## Conclusion

This study investigated deglutition during sleep in patients with OSA, with a focus on respiratory arousal. A negative correlation between AHI and deglutition during shallower sleep was found.

## Figures and Tables

**Table1:** The average index of deglutition

The average±SD	Deglutition with arousal	Deglutition without arousal	Overall
Sleep-stage I	5.4±3.7	4.3±3.5	9.7±6.0
Sleep-stage II	1.2±3.1	0.7±1.4	1.9±3.7
Rem-sleep	4.7±6.5	1.6±2.7	6.3±6.7
All sleep	3.4±2.5	2.3±1.5	5.7±3.1

REM: Rapid Eye Movement Units: Times per hour

**Table2:** Correlation coefficient between the “index of deglutition” in the total sleep stage and each sleep parameter

	Deglutition with arousal	Deglutition without arousal	Overall
AHI CC (Pv)	–0.17 (0.41)	–0.24 (0.24)	–0.25 (0.22)
AI CC (Pv)	–0.13 (0.53)	–0.22 (0.30)	–0.21 (0.32)
HI CC (Pv)	–0.06 (0.77)	0.13 (0.53)	0.02 (0.94)
Lowest SpO_2_ CC (Pv)	–0.13 (0.53)	0.32 (0.12)	0.05 (0.80)

AHI: Apnea Hypopnea Index, AI: Apnea Index, HI: Hypopnea Index, CC: Correlation Coefficient, Pv: P-value

**Table3:** The correlation with other sleep stages and AHI

	Deglutition with arousal	Deglutition without arousal	Overall
Sleep-stage I CC (Pv)	–0.33 (0.01)	–0.48 (0.01)	–0.48 (0.02)
Sleep-stage II CC (Pv)	0.12 (0.55)	0.08 (0.71)	0.13 (0.52)
Rem-sleep CC (Pv)	–0.17 (0.42)	0.54 (0.10)	0.05 (0.80)
All sleep CC (Pv)	–0.17 (0.41)	–0.24 (0.24)	–0.25 (0.22)

AHI: Apnea Hypopnea Index, REM: Rapid Eye Movement, CC: Correlation Coefficient, Pv: P-value
